# How harmful is right ventricular pacing? The question revived by the BioPace trial

**DOI:** 10.1093/europace/euaf005

**Published:** 2025-03-19

**Authors:** Mads Brix Kronborg, Niraj Varma, Jens Cosedis Nielsen

**Affiliations:** Department of Cardiology, Aarhus University Hospital, Palle Juul-Jensens Boulevard, Aarhus 8200, Denmark; Department of Clinical Medicine, Aarhus University, Aarhus 8200, Denmark; Department of Cardiovascular Medicine, Cleveland Clinic, Cleveland, OH 44118, USA; Department of Cardiology, Aarhus University Hospital, Palle Juul-Jensens Boulevard, Aarhus 8200, Denmark; Department of Clinical Medicine, Aarhus University, Aarhus 8200, Denmark

This issue of *Europace* presents the results of the Biventricular Pacing for atrioventricular Block to Prevent Cardiac Desynchronization (BioPace) trial. The main findings of the completed BioPace trial were presented as a late breaking clinical trial and abstract over 10 years ago. Now, the full data are available to review in a well-written manuscript.^1^ Provocative questions emerge.

The BioPace randomized controlled trial tested whether cardiac resynchronization therapy (CRT) with biventricular pacing was superior to right ventricular (RV) pacing (predominantly apical) in patients with atrioventricular (AV) block expected to receive a high burden of ventricular pacing. The trial was large, enrolling 1810 patients at 94 medical centres in 15 countries. During a mean follow-up of >5.5 years with trivial patient attrition, there was no significant difference between the two treatment groups with respect to the two co-primary endpoints: *the composite of time to death or first heart failure hospitalization* and *survival time*. Nor was any difference observed in 6-min hall walk test or quality of life after 2 years between treatment groups. Most patients had normal left ventricular ejection fraction (LVEF) at baseline. However, results were unaffected by LVEF stratification, i.e. normal, reduced (36–50%), and low (≤35%) LVEF. However, there were only 152 patients with LVEF < 35% underpowering this comparison.

This trial was the first large-scale randomized controlled trial investigating whether CRT reduces heart failure and deaths in patients with AV block committed to cardiac pacing. The results reject that hypothesis. It is well documented that RV pacing in patients with severely reduced LVEF and heart failure worsens heart failure and reduces survival.^2^ In such patients, avoidance of ventricular pacing (when possible) and CRT implantation are better choices. However, the picture is less clear in patients with preserved or mildly reduced LVEF, although observational data have supported that RV pacing in these patients also may cause heart failure.^3,4^ The BLOCK-HF trial showed a benefit of CRT in patients with LVEF <50% and AV block where almost 70% had a LVEF ranging from 35 to 50%. This was primarily driven by left ventricular remodelling, although CRT also reduced hospitalization for heart failure.^5^ However, BioPace presents important clinical endpoints questioning how harmful RV pacing is in patients with AV block and preserved or mildly reduced LVEF. The patients in BioPace had close to 100% ventricular pacing in both groups during the entire study period. The finding of no benefit from CRT over RV pacing is concordant with other large, randomized trials where preservation of AV synchrony in AV block^6^ or sinus node dysfunction^7^ or avoidance of RV pacing using atrial based pacing (AAI) pacing in sinus node dysfunction^8^ did not improve survival or reduce heart failure. These trials were conducted mainly in patients with preserved LVEF.

The BioPace results reopen the discussion about pacing-induced heart failure, itself a term lacking a consensus definition. It may be best described as a poorly understood disease state occurring in a minority of patients treated with cardiac pacing manifesting with LV dysfunction and sometimes clinical heart failure. Observational data from the Danish National registries indicate a cumulative incidence of heart failure of 10.6% after 2 years in patients treated with cardiac pacing.^9^ The excess risk dominated during the first months after pacemaker implantation, then declined to marginal values beyond 6 months.^9^ A recent Swedish registry reported a heart failure incidence of 21% after 5 years and identified nine risk factors including higher age, male sex, chronic kidney disease, atrial fibrillation, and chronic obstructive lung disease. Surprisingly, AV block was a lesser risk.^10^ Thus, pre-existing myocardial disease and/or accompanying comorbidities may determine the development of heart failure during RV pacing. Interestingly, RV pacing prolongs QRS most in patients with LV dysfunction.^11^ While RV pacing in patients without LV dysfunction causes an abnormal ventricular activation and a 5–7% decrement in LVEF,^12,13^ it remains unknown whether this action *per se* causes heart failure or whether, in some patients, a parallel myocardial disease process exacerbates the tendency to develop pacing-induced HF.

BioPace publication times fortuitously with current debate regarding best pacing prescription. Conduction system pacing (CSP) has been introduced as an alternative to traditional biventricular pacing and advocated to prevent pacing-induced heart failure, particularly in those patients anticipated to have a high proportion of ventricular pacing.^14–17^ BioPace results indicate that harmful effects of RV pacing develop infrequently among patients with preserved LVEF, both when measured by survival and by softer parameters as functional capacity and quality of life. Safety is important. In BioPace, more lead-related complications were observed in the CRT group than in the RV pacing group during follow-up. We know that AV synchronous RV pacing is an excellent treatment of AV block, effectively protecting the patients against syncope and sudden death, associated with device battery longevity exceeding a decade in the current era, and with few long-term complications. Although CSP tends to yield narrower QRS complexes^18^ and slightly better echocardiographic ventricular function^19^ compared to RV pacing, whether this translates into superior (or even non-inferior) long-term patient outcomes (survival, heart failure, functional status, and quality of life) is uncertain. Long-term complications are unknown. Therefore, it may be more appropriate to use RV pacing when LVEF is preserved or mildly reduced and upgrade the few patients who actually manifest pacing-induced heart failure, understanding that this entails additional risk and costs. This can be a shared decision.

There are several non-negligible limitations in the BioPace trial. The data are at least 10 years old. However, BioPace was a well-conducted randomized controlled trial with 1810 patients, >700 primary outcome events, a follow-up period relevant to cardiac implantable electronic device treatment, and capture of chronic effects of pacing modes and treatment complications. Changes in design components of the trial—change in primary endpoints, increase in sample size, and change in follow-up time—were made during the course of the trial. However, these were all appropriate, reflecting well thought-out and necessary changes (appropriately explained in the supplement). The (13%) failure rate of successful left ventricular lead implant at first attempt among patients randomized to CRT was greater than expected (no repeat attempt was made in a considerable proportion). This may result in a lower-than-expected difference between treatment groups. It is unknown whether establishment of biventricular pacing for all patients in the CRT group would have changed the results. However, implanters were advised to place the left ventricular leads in a coronary sinus branch in a posterolateral position, which is considered the best choice also today. Furthermore, an ‘as treated’ analysis confirmed no advantage of CRT vs. RV pacing.

Finally, no echocardiographic data were reported: changes in LVEF and left ventricular size may have provided insights into the clinical findings.

The BioPace investigators should be congratulated on finalizing and now also—despite an unusual delay—reporting this well-conducted large, randomized trial. The data add significantly to current knowledge and must be considered when discussing the best pacing mode for patients with AV block—CSP, CRT, or RV pacing? Contemporary practice has been influenced by observational data sets, but these should be considered potentially flawed by selection bias and confounding irrespective of statistical adjustments. BioPace findings reinforce that well-designed and carefully conducted randomized controlled trials set the standard to guide changes in clinical practice.
